# Making it work for me: beliefs about making a personal health record relevant and useable

**DOI:** 10.1186/s12913-018-3254-z

**Published:** 2018-06-14

**Authors:** Fiona Fylan, Lauren Caveney, Alastair Cartwright, Beth Fylan

**Affiliations:** 1 Brainbox Research, 46 Town Street, Gildersome, Leeds, LS27 7AA UK; 2 Leeds Sustainability Institute, Leeds Beckett University, Leeds, LS1 3HE UK; 3 NHS Leeds North Clinical Commissioning Group, Leafield House, 107-109 King Lane, Leeds, LS17 5BP UK; 4 School of Pharmacy, Faculty of Life Sciences, University of Bradford, Bradford, BD7 1DP UK

**Keywords:** Personal health record, Electronic health record, Health communication, Patient-centred care, Patient acceptance, eHealth

## Abstract

**Background:**

A Personal Health Record (PHR) is an electronic record that individuals use to manage and share their health information, e.g. data from their medical records and data collected by apps. However, engagement with their record can be low if people do not find it beneficial to their health, wellbeing or interactions with health and other services. We have explored the beliefs potential users have about a PHR, how it could be made personally relevant, and barriers to its use.

**Methods:**

A qualitative design comprising eight focus groups, each with 6–8 participants. Groups included adults with long-term health conditions, young people, physically active adults, data experts, and members of the voluntary sector. Each group lasted 60–90 min, was audio recorded and transcribed verbatim. We analysed the data using thematic analysis to address the question “What are people’s beliefs about making a Personal Health Record have relevance and impact?”

**Results:**

We found four themes. *Making it work for me* is about how to encourage individuals to actively engage with their PHR. *I control my information* is about individuals deciding what to share and who to share it with. *My concerns* is about individuals’ concerns about information security and if and how their information will be acted upon. *Potential impact* shows the potential benefits of a PHR such as increasing self-efficacy, uptake of health-protective behaviours, and professionals taking a more holistic approach to providing care and facilitating behaviour change.

**Conclusions:**

Our research shows the functionality that a PHR requires in order for people to engage with it. Interactive functions and integration with lifestyle and health apps are particularly important. A PHR could increase the effectiveness of behaviour change apps by specifying evidence-based behaviour change techniques that apps should incorporate. A PHR has the potential to increase health-protective behaviours and facilitate a more person-driven health and social care system. It could support patients to take responsibility for self-managing their health and treatment regimens, as well as helping patients to play a more active role when care transfers across boundaries of responsibility.

## Background

A Personal Health Record (PHR) is an electronic record that individuals can use to help manage and share their health information. One of its aims is to encourage individuals to take more individual responsibility for their own health by becoming more engaged in the health care process and by improving communication between individuals and their healthcare providers [1]. PHRs have been developed both by healthcare providers (e.g. HealthSpace) and private organisations (e.g. Google Health and Microsoft’s HealthVault). There are several models of PHR, with some being more clinician-focused than patient-focused, and some are standalone rather than tethered to the individual’s medical records [1]. Both healthcare professionals and patients prefer systems that are linked to medical records [2, 3]. However, PHRs are distinct from electronic health records (EHRs) as they allow contributions from patients rather than solely collecting medical information following interactions with health services [4], and there is only one PHR whereas there may be several EHRs for the same patient [5]. A taxonomy for PHRs has recently been developed [5] that specifies a PHR in terms of its: architecture (how data is stored and geographical coverage); structures (the type of data it contains and the data standards it uses); and its functions (who accesses it, who can edit it, who inputs data, and whether its goal is for patients to consult, maintain or monitor their data). Common PHR functions are to request repeat prescriptions, make appointments, view and update medication and allergy details, view test results, communicate with healthcare providers [6] and (less commonly), make lifestyle changes [7]. Some patients also want a social network function that enables them to make contact with others with the same condition [8]. Because of this focus on PHRs to monitor health conditions, younger and healthier people are less likely to find them useful [2].

There is evidence that having a PHR is beneficial for health, affording individuals greater control over their own health and wellbeing and helping them to better manage their health conditions [9]. There is evidence that a PHR can improve outcomes in several conditions, including asthma, diabetes, fertility, glaucoma, HIV, hyperlipidemia, and hypertension [10]. A PHR can increase uptake of screening tests and increase adherence to medication [11, 12], reduce the number of consultations people have with their doctors [13] and enable people to access support when their condition deteriorates [14]. However, several studies implementing a PHR have found no positive effects on health outcomes and it has been suggested that poor patient engagement with PHRs may explain the lack of improved health outcomes found [15, 16]. When patients have low expectations of the personal benefits of using and maintaining a PHR this hinders engagement with the record [7].

Patients’ concerns about a PHR include privacy and security, usability and relevance [7, 17] and there are several barriers to using one [18, 19]. People can be uncomfortable accepting recommendations from a software programme – particularly when the advice they receive does not appear to be tailored to them personally – and they prefer to have some degree of interaction with healthcare professionals [2]. While clinicians believe PHRs can improve information sharing and communication between themselves and their patients, they have several concerns about using them, including increased workload, the potential to confuse patients or alienate people who do not use them, and increasing health inequalities through the ‘digital divide’ [20].

While we know about the beliefs and experiences that individuals with existing health conditions and their clinicians have about a PHR, there is little research that explores younger and healthier individuals’ interest in and expectations for a PHR, including what functions would make it more personally relevant. This research explored beliefs about PHRs held by people with many different experiences, including teens, older adults, physically active adults and people with health conditions. We aimed to capture individuals’ ideas for how they could and would use a PHR, their concerns, the potential impact it could have on how people perceive health and illness and their willingness and ability to take personal responsibility for their health.

## Methods

We adopted a qualitative approach, comprising focus groups which were analysed thematically (Braun and Clarke [21]). This approach was essential to provide sufficient insight into individuals’ beliefs about and expectations for a PHR.

### Focus groups

Focus groups enabled dynamic discussions about how a PHR could be used. Each focus group lasted around 90 min and, with the permission of participants, was audio recorded. All focus groups were facilitated by one of the authors (FF) with an assistant taking notes. The facilitator introduced the topics for discussion, ensured discussions stayed on topic, and ensured that all participants contributed. We developed a semi-structured focus group topic guide with a series of questions, probes and prompts that allowed us to elicit participants’ views and then explore them in a meaningful way. Each focus group explored the following areas, with questions and the language used tailored to each group:

- Beliefs about the value of having a PHR.
- How people would anticipate using a PHR.
- The information people would expect to see in their PHR.
- The information they might want to contribute to their PHR.
- Beliefs about how a PHR might affect personal responsibility for health.
- Which professionals they anticipate interacting with their PHR.
- Concerns around having a PHR.

Table 1 shows which aspects of the PHR taxonomy [5] patients were asked about, and which aspects were specified for them.

**Table 1:** Aspects of the PHR discussed during focus groups

Group and item	Approach
Structures	
Data type	Discussed during the focus groups.
Standards	Neither discussed nor specified.
Functions	
User profiles	Discussed during the focus groups.
Interaction	Specified as direct (patients own and manage data in their PHR).
Data source	Discussed during the focus groups.
Goals	Discussed during the focus groups.
Architecture	
Model	Specified as inside: the PHR is stored by the provider.
Coverage	Discussed during the focus groups.

Groups commenced with a description of the project, the opportunity for participants to ask questions, and then each gave informed consent to take part. Parents of the under 18 s also gave informed consent for their child to take part. The following explanation of a PHR was given to each of the groups and participants could ask questions to clarify their understanding:

*The NHS and the City Council in our city are thinking about developing Personal Health Records that people can use to manage and plan their health and wellbeing. You would have your own record and you could put whatever information you want in there – whatever is relevant to you. It’s separate from your medical records (hospital, GP, dental, physiotherapy,* etc.*) but potentially you could use it to add notes to your medical records or include information from your medical records.*

### Participants

Our sampling strategy was to include people with a range of different experiences that might affect how they respond to and interact with a PHR. We included adults with existing long-term conditions as well as those without health problems. We included older adults, physically active adults, young adults and also teens, as they might want to use a PHR to log different aspects of their health and wellbeing to adults. We also included professionals working in a role that involves using data to benefit services and wider society. Finally, we included professionals from the voluntary sector who provide advocacy for people with a range of health conditions. We had no inclusion or exclusion criteria based on medical knowledge or computer literacy so each group contained participants who varied in these aspects. More details of the composition of the focus groups are shown in Table 2.

**Table 2:** Composition of focus groups

Group	Composition and characteristics
FG1	Six participants who all had one or more long-term health conditions including: diabetes, coronary heart disease, asthma and mobility problems.
FG2	Eight participants all with long-term health conditions. In addition to their own health problems, two participants were carers for relatives.
FG3	Seven young people aged 14–18. They were all at school or at college. None had any health conditions.
FG4	Seven young adults aged 18–24. They were either studying or working. None had any health conditions. Discussions in this group also explored the health concerns young adults have and the transition to being responsible for their own health.
FG5	Seven older adults, aged 60–85. Discussions in this group also included willingness to learn to use new technologies.
FG6	Seven amateur triathletes who all use devices to monitor their health and training. Discussions in this group also explored how they could use the data they collect to benefit their health and healthcare.
FG7	Seven participants, all with a professional interest in data use. This group also explored how future developments in technology might affect how people could use a PHR.
FG8	Six voluntary sector workers who represented organisations providing advocacy services for people with a range of disabilities and physical and mental health conditions. These participants discussed how they would personally use a PHR, how the people their organisation work with might use one, and how their organisation might interact with their service users’ personal health records.

We analysed the data using thematic analysis according to the methods of Braun and Clarke [21]. The data were broken down into codes which were then grouped into areas of similar meaning (sub-themes), and the sub-themes grouped into themes which are internally coherent, consistent, and distinctive and which address the research question. The research question we used in the analysis was “What are people’s beliefs about making a Personal Health Record have relevance and impact?” One researcher coded the transcripts and sorted the codes (FF) and a second researcher (LC) reviewed the codes. Researchers FF and LC organised themes and subthemes and all authors reviewed the final thematic structure against transcript extracts.

## Results

**Fig. 1:**
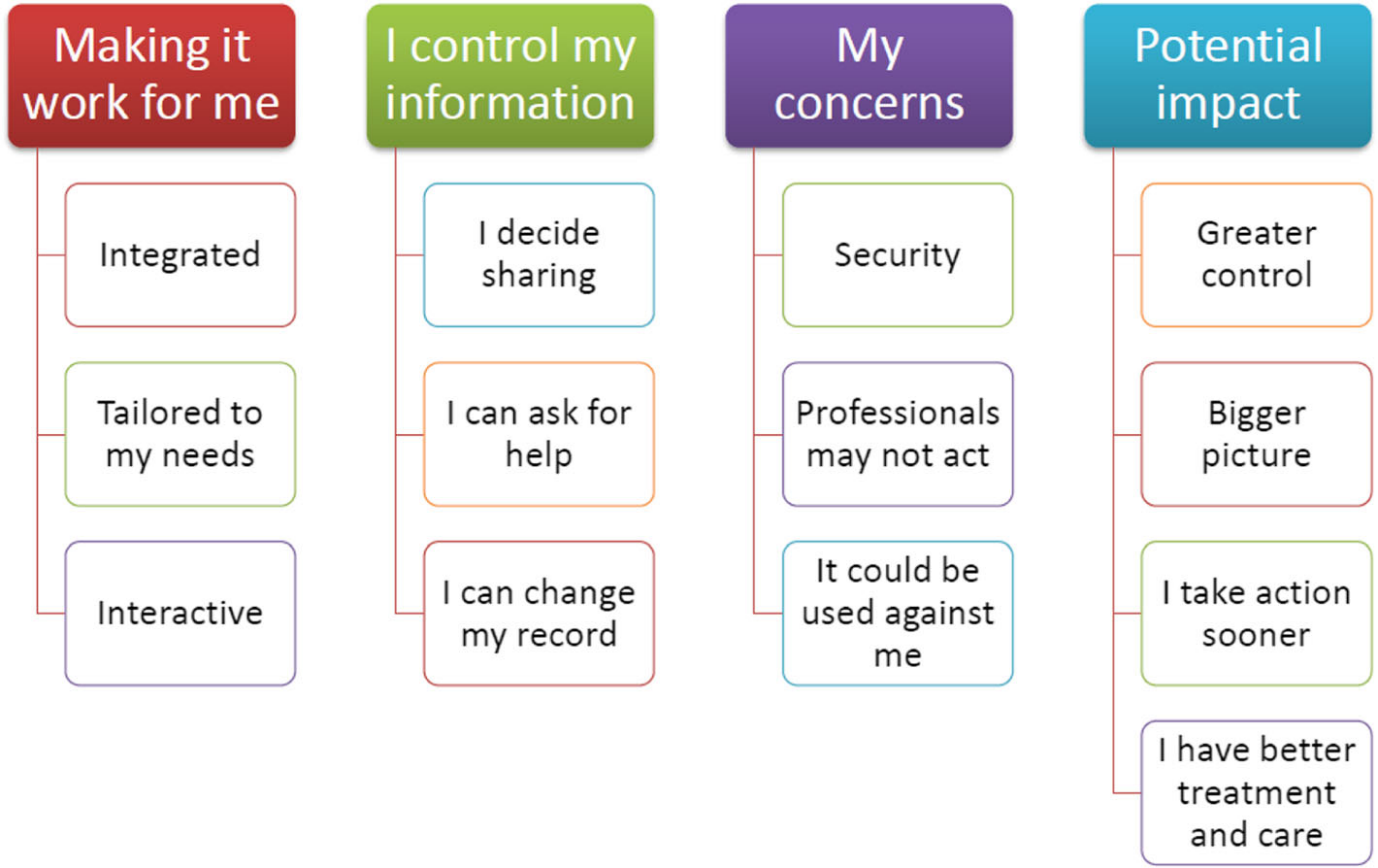
Thematic map of what people think about a personal health record

We identified four themes in the data: making it work for me; I control my information; my concerns; and potential impact. Each of the themes comprised three or four sub-themes which are displayed in Fig. 1. The themes and sub-themes are described below and illustrated with verbatim quotes from selected focus groups, chosen by the research team to be representative of the data.

### Making it work for me

This theme is about how a PHR could be developed to encourage people to actively engage with it. There are three subthemes: integrated; tailored to my needs; and interactive.

### Integrated

Participants talked about the importance of a PHR being integrated with other professional records and apps to make it easy to use, engage with and keep up to date. They wanted the ability to pull information from other sources rather than having to enter it themselves. This included information tethered to their National Health Service (NHS) records such as details of appointments and information on medication, and also information collected by fitness monitoring apps such as Strava, which allows users to track and analyse fitness activity, and map and share routes with other users. Participants explained how it can be difficult to remember all the names and dates of their conditions, vaccinations and medications and so a record that automatically pulls this from their medical records would be valuable. Some participants talked about how they already keep paper or electronic health records and they would appreciate having a PHR because it would make it easier for them to enter, store, extract and share information. They discussed how a PHR could provide a home for all the information they collect and enable them to use it in an integrated way to improve their health and wellbeing.

*“It needs to be up to date, especially if it has health information and medication records in it. If you’re leaving it up to the individual, sometimes people get confused about what medication they’re on and what dosage.” (FG8)*

*“My son is diabetic and we write down what we feed him, what his insulin is, what his bloods are. It would be great if there were an app for writing that down.” (FG2)*

*“Since I have got older I have more ailments and I do forget what I have had, especially with the menopause. I wish I had written things down.” (FG1)*

*“At the minute I’ve got Strava, [My]Fitness Pal [an app to track calorie intake and exercise], I’ve got seven or eight different apps and my data’s just all over the place. So having one place of truth might make it something that I use a lot more often and put more data in there.” (FG6)*

Participants discussed how there could be a range of apps developed to help make keeping an accurate record easy, for example to track their physical activity or rate their mood, or scan barcodes from over-the-counter medicines or the food they eat.

*“There’s a potential to barcode scan, you know, you buy ‘Lemsip’ [a cold and flu remedy] and you pop it in because then all the other little ingredients that you don’t realise are there, if that causes an allergic reaction.” (FG8)*

### Tailored to my needs

Participants identified a wide range of information relevant to their health and wellbeing that they would want to keep a record of. However, they wanted the PHR to be tailored to their own requirements. For example, some people wanted to keep daily records of measurements such as heart rate and blood pressure but recognised that these functions should be optional. Those with a more complicated medication regimen suggested having an app to remind them to take their tablets but recognised that not everybody would want to use this function. Nevertheless, there was a core set of common information that participants believed it would be useful to keep for everybody, such as medication, conditions, appointments and test results.

*“If you’re taking medication ever day at regular times, you think ‘Did I take my tablet at 2pm? Did I not?’ Well, if you put it in [your PHR] you could look back and check.” (FG2)*

Participants also discussed how the things that are relevant to their health and wellbeing change over time so they would like to be able to change what they record accordingly. They talked about how the PHR may seem less important when you are healthy so you may not want to interact with it very much. However, if you are diagnosed with a condition there are a lot of things that become important to find out about and monitor and the PHR should support this varied level of input. Participants discussed how they might want to allow their GP, other health or social care professional, their employer or their school to access something in their record while a condition is being investigated or treated. However, they didn’t believe access should be permanent; they wanted flexibility to turn permissions on and off whenever they believe appropriate, including if they move to a new employer or school.

*“Whilst you’re healthy you take things for granted but when you develop a long-term condition then I think that could be quite a crucial thing in terms of a reflective piece about how a treatment’s working or not.” (FG8)*

*“You’re giving a bigger picture so they’ve got a better understanding of your needs, but after that consultation it’s gone. So you can say, ‘Right, from this point on I don’t want you to have that information anymore because I’m no longer being treated for that,’ or, ‘I’m no longer receiving that particular care’.” (FG8)*

### Interactive

This sub-theme describes participants’ discussions around the PHR being able to send them notifications of things they might be interested in, or alerts to warn them when a particular set of their measurements indicates they need to consult a health professional. Some talked about how the PHR could help them measure and monitor their health behaviours and outcomes (e.g. by graphing their weight against the amount of exercise they have done) and send them messages about their progress or to encourage them to maintain or change their behaviour. Some talked about how they would like to send a message to a professional to flag up an aspect of their PHR they are concerned about.

*“For me, health is all about my weight. I’ve started a running club with two friends and it would be good to get messages to say how far I’ve run.” (FG5)*

*“On the watch you have these little circles that go around and it’s kind of, I want to complete these circles every day. It motivates you to be bothered.” (FG6)*

Some participants suggested having a section of their PHR that they could use in a way similar to social media to join online communities and share information, experiences and advice with others.

*“It would be useful for me to contact other people with diabetes to maybe ask a particular question or to hear other people’s experiences.” (FG2)*

### I control my information

This theme is about how a PHR should enable individuals to control their own information and decide who has access to which information. There are three subthemes: I decide what is shared; I can ask for help; and I can change my record.

### I decide what is shared

Throughout all the focus groups participants highlighted the desire to choose to share whatever information they want with whoever they want. Participants discussed how some information, for example about sexual or mental health, is more sensitive and they would want to keep this in a separate section of the PHR that is not accessible to medical professionals unless they specifically granted permission. Participants also wanted to control sharing with family members, for example so they could find out whether there is a family history of a particular condition.

*“I want the ability to let whoever I want to have access to my PHR whenever I want.” (FG7)*

*“People can know about my cancer but my mental health issues – I’m not so willing.” (FG1)*

Participants who had experience of trying to co-ordinate healthcare across multiple agencies described how it can be very frustrating, as well as costly, that different healthcare systems do not share information. They talked about how having a PHR would mean that they become the information conduit between different agencies. In this way the information boundaries between the NHS, local authority, private companies, and front line professional would all disappear. Participants talked about how this would make caring for themselves or others much easier.

*“I would want to use it to tell my story once. So if I’ve been involved in a really serious car accident I can put that on there and it doesn’t matter which organisation or hospital or doctor I’m seeing, they know that I’ve had a serious head injury, etc, and I don’t have to keep telling that story every time.” (FG8)*

*“I go to Leeds for a lot of my healthcare, I go to York for my eyes, which are related to my thyroid and when I get there they want to know what my thyroid’s doing but my thyroid’s been tested in Leeds. So if there was a record that I have I could say ‘This is my last blood test results.’ it would save York having to do another blood test.” (FG2)*

### I can ask for help

Participants believed a major benefit of the PHR could be that they can send information to their GP or other health or social care professionals to ask for advice or query whether they need an appointment. This included both physical and mental health. Participants discussed how tracking mood in a PHR might mean they recognise they need to seek help for depression and the information they had collected would make it easier to talk to a professional about how they are feeling. Some participants from the teens focus group talked about how they could use an interactive PHR to seek help with mental health concerns: they might not know where to go for help or feel unable to talk to their GP but they could seek help from an NHS online community.

*“Some people might use it to get help. They might want help but they don’t know where to go.” (FG3)*

*“It would only make a difference if somebody was looking at the information, if you could send it to a doctor or somebody and who would contact you to let you know if you needed to do anything.” (FG4)*

### I can change my record

Some participants talked about wanting to delete aspects of their record, for example when they believed that an old condition or experience was no longer relevant to their current or future health needs. There were discussions around whether participants should be able to delete any professional sections on their PHR. Some participants believed that if this is a truly personal record they should be able to control its content, while others highlighted that only medical professionals are truly qualified to decide whether conditions or treatments remain relevant. Therefore, some believed it would be better for individuals to annotate the record rather than delete parts. Some participants talked about how information on medical records can be inaccurate and a PHR would make it more likely that they spot errors and ask for them to be corrected.

*“Things in your life may have changed and you don’t want it seen in the future because you’ve moved on from there. From this point I don’t want you to have that information because I’m not being treated for that anymore.” (FG8)*

### My concerns

This theme is about the concerns that individuals have about a PHR and how they could be reassured. There are three themes: security; professionals may not act; and it could be used against me.

### Security

Participants discussed how secure their records would be and they thought that PHRs are at risk for three reasons. First, there could be a breach of security at NHS level, in which all records are leaked. Some participants were bemused by why this might cause them a problem but a few, all with professional IT experience, described how this might lead to identity theft. Second, some participants expressed concerns around the individual’s own PHR being accessed by people who know them, for example, if others observe them entering or find a written note of their login details or they are coerced into giving somebody else their details. Adults discussed how they might be expected to share details with a partner yet might not want to. Younger people described how they could be bullied at school if others found out about their records. Third, participants discussed concerns that their information could be sold, either by the NHS or by private companies they share their PHR with. Participants were concerned about being contacted by commercial companies trying to sell them treatments or equipment for their condition. Participants who work in the voluntary sector were concerned about security implications for their clients but less so for themselves personally.

*“We’re living in a cyber world where large companies with additional safety and security measures in place get hacked. I think people would back away from having data on their health record if there was a chance of it getting hacked and there’s always going to be that chance.” (FG8).*

During the focus groups participants were asked about the potential for information from their PHRs to be combined with those of others and used for research purposes. Nearly all were happy for this to happen, providing that their data were anonymised. Some participants preferred for their data to be only used by NHS researchers, and others did not want it to be used by pharmaceutical companies, while others were happy for it to be used for any research.

### Professionals may not act

Participants had several concerns about how they would know whether a professional had received any messages they had sent, or reviewed some aspect of their PHR. They suggested that the PHR should log when the information had been sent and when it had been opened. This way they would feel reassured that their query or their case had not been forgotten about.

*“You need to have something on there that tells you that your record has been read and an email that comes through and says ‘There is an answer waiting for you on your personal health record’.” (FG8)*

Some participants were sceptical about whether or not their GP or other healthcare professional would trust the records they kept, or be able to understand the information. However, they talked about how they could select a professional based on their interest in using the patient’s PHR. For example, a private physiotherapist could track the information collected by the apps to explore in detail how an individual is recovering from an injury and responding to treatment.

*“I had an issue a couple of years ago, backwards and forwards to the doctors and I was fobbed off. Eventually I saw a nurse who recommended I see a specific doctor because he did some running. I took the information from my Garmin [a device with functions of global positioning, activity tracking and heart-rate monitor pairing] which showed VO2 [maximum oxygen uptake] going downhill. We had a sensible conversation, somebody who understood what I was saying. I came out with the right course of antibiotics and five days later I was fine.” (FG6)*

### It could be used against me

During the focus groups participants discussed concerns about how their PHR could be used against them. For example, they wondered whether an employer might insist on viewing their PHR before making them a job offer or an insurance company before insuring them. A few participants discussed whether or not the NHS could use information from their PHR to decide whether or not a treatment would be offered on the NHS. They wondered whether lifestyle information could be used to decide whether a condition was “self-inflicted”, in which case, any treatment might not be funded.

*“You go for a job. ‘Oh yes, can we have a look at your personal health record?’ And you might have put down that you’re feeling depressed for three days and they might say ‘We don’t want him’.” (FG 2)*

*“The worst thing I can think is insurance companies getting hold of personal health records and then deciding ‘We’ll insure you for this but not for that because your grandfather had it.” (FG5)*

*“If people are more responsible for their health, and equally then, could the NHS turn round and say ‘Well yes, that’s self-inflicted, so you want that operation to staple your stomach? You’re paying, not Mr Taxman’.” (FG1)*

### Potential impact

This theme is about participants’ ideas for the potential impact that the PHR could have on people’s health and behaviour and the health and social care system. There are four sub-themes: greater control; bigger picture; I take action sooner; I have better treatment and care.

### Greater control

Participants discussed how a PHR would make them feel they have more control over their own health and wellbeing. This would arise from being able to see and interact with their own records, actively tracking their health measures, checking for test results, or requesting advice from healthcare professionals. Their records would contain information that they believe is personally relevant to them. For many, this would mean that they take greater responsibility for their own health. For example, they might track lifestyle changes they make to help manage a health condition. The PHR would make it easier for them to identify and recall whether the changes are working.

*“You might want to keep track of diet and exercise and see if there are any changes. You might want to go back over a long time. I’ve done that myself and tried to remember whether something I tried two years ago actually had an effect but I can’t remember if it worked.” (FG2)*

Some participants highlighted how the PHR might help the NHS become less paternalistic. A PHR might increase the culture of shared responsibility for preventive health and for treating conditions. They discussed how being able to make notes about their condition would mean that they and their healthcare professional are more able to assess whether treatment is effective. This might lead to more open discussions between individuals and professionals about expectations and aspirations for treatment and how they are responding to treatment. In this way ineffective treatments are halted sooner and individuals would be more likely to take their medications as agreed.

*“In the US you’re treated as an equal and it’s like ‘How are we going to solve this together?’ whereas in the UK there’s either not the time to do that or it’s just not culturally the norm.” (FG7)*

### Bigger picture

Participants believed that GPs and other healthcare professionals could use information from the PHR to build a more holistic picture of how an individual’s condition and lifestyle affects their health and wellbeing. The PHR could give professionals greater insight into an individual’s behaviours that promote and risk health. This could lead to greater discussion around treatment options and medicines regimens and this could help to optimise medicines. Participants thought that having access to this wider set of information could help GPs better identify needs and to make greater use of social prescribing.

*“It’s going to be beneficial to have a more complete picture. Not just of someone’s health, things that have gone wrong, but what they are doing day-to-day.” (FG6)*

*“This seems much more of a preventative, effective way. GPs can do, what do they call it, social prescribing, where they say ‘You could do with a bit of exercise, there’s a walking group here and you can go and do that’.” (FG2)*

Participants also discussed how the PHR could include large amounts of information from current and future technologies such as wearable health and fitness monitoring devices. They talked about how this technology could help people live independently for longer, for example if it were monitored by professionals and/or family members.

### I take action sooner

Participants discussed how they might use a PHR to identify and respond to problems sooner. By monitoring certain aspects of their health they would notice when problems arise or their condition is deteriorating before it causes health difficulties. They might take action to reduce their risk of developing a condition, or to better control their existing condition. Alternatively, they might seek help for any problems they identify and prevent a condition from arising or get treatment at an earlier stage. They could base these decisions on aspects of their health and lifestyle that are relevant to them as individuals.

*“It could mean that you start thinking about what you have to do to stay healthy. About exercising more.” (FG2)*

*“If you’re just seeing little bits you don’t really do anything about it but if you have it all together you can maybe see where you are going wrong. Like people with anorexia, if the GP had access to that information before you went to see him then maybe they could do something about it before it turns into a secret and you’ve got to hide it.” (FG3)*

Some participants talked about their PHR giving them a health score to indicate current health and risk of future health conditions. They talked about how the PHR, or related apps, could make suggestions of things they can do to increase their health score.

*“Maybe have, like warnings, stop it before it happens, Like if it was to do with weight, one day I’m seven stone [a ‘stone’ is a unit of measurement equal to 6.35kg], then it’s eight stone, if you’ve got a graph you’re going to see that you need to sort that out, then there’s a section where you can go and get help.” (FG3)*

### I have better treatment and care

Participants believed that access to better and more complete information would help ensure that people receive better treatment and care. Sharing information from their PHR with a range of professionals, including health and social care professionals, would mean that these professionals are better informed about the individual’s complete care package. Participants discussed how it can be very difficult to give healthcare professionals a full account of their medical history yet they are sometimes expected to do so. Also, because individuals could use their PHR to give healthcare professionals a more accurate account of symptoms and how their condition has changed following treatment, it is likely that diagnosis and treatment is more appropriate.

*“If you’ve got an appointment with the physio, the first thing they say is ‘What’s wrong with you?’ and if the doctor’s sent you there you can’t explain it, in medical terms, and they seem to expect that you’ve memorised the exact terminology for the exact bone, muscle, whatever.” (FG6)*

One participant talked about his fears of having another stroke and being unable to communicate his medical history and care preferences. He believed that a PHR would ensure that the records he has been keeping, together with his wishes, were taken into account. Similarly, other participants talked about using their PHR to record their end-of-life wishes.

## Discussion

We have identified four themes that describe what people think about having a PHR, how they anticipate using it, and the difference they expect it could make to people’s health and wellbeing. They highlight how a PHR could be made more personally relevant for people and the results provide a blueprint for designing a PHR that will engage users. Participants wanted the PHR to be interactive, sending them messages, enabling them to make contact with others in a similar situation, and also to communicate with their healthcare professionals, which supports previous findings [14, 22]. In addition, we found that people want the PHR to be tailored to their needs and capable of changing over time, alongside changes in their health and wellbeing needs. They saw it as a way of storing information relevant to their health and wellbeing, monitoring the effectiveness of both formal and self-directed interventions, and sharing their information with others, such as professionals, carers and family members.

Participants wanted control over their own PHR, including who they share information with, and to use their PHR to request help or advice from a healthcare professional. Indeed, participants thought the main value of a PHR was the ability to share the information it contains with others and to circumvent problems arising from lack of connectivity between different health and social care record systems. In the UK, there are IT connectivity challenges which mean that information from different hospitals, and between primary and secondary care, cannot be shared. There are also challenges in sharing data between NHS and commercial providers. Previous research has found that doctors can be resistant to the idea of a patient-held record as they do not believe patients are capable of using them [23] but our participants with a long-term condition were keen to learn more about their condition and how to manage it.

Participants’ discussions provided insight into how the PHR should be described so that people find it more relevant. They wanted the PHR to enable a more holistic and less paternalistic health and social care system. They believed that being able to see and interact with their own records, actively tracking their health measures, checking for test results, and requesting advice from healthcare professionals would give them greater control over their own health and wellbeing and would encourage health-protective behaviours and discourage risky health behaviours in both healthy individuals and those with a health condition. Previous research has focused on the beliefs and experiences of a PHR in people with a specific health condition [10], so our approach to include healthy individuals has added new knowledge about how to encourage greater interest in a PHR and how to maintain health and wellbeing using more health-preventative activities. In this way, the results are widely transferable. However, our participants all lived in a single city in the north of England, and while we included people with a range of ethnicities, all could read and write in English. People whose English is not as proficient may have different perspectives.

Though it has been suggested that people are reluctant to use technology to access their PHR [24], we did not find evidence of this. Participants were concerned about security breaches, primarily about their PHR data being sold to commercial companies and resulting in them receiving sales calls, as identified previously [17]. We also identified new concerns: that their PHR might disadvantage them in the future, for example if potential employers and insurers asked to see their record, and whether the NHS healthcare system might deny them treatment because their condition is lifestyle-related. They were not worried about not being able to understand information in their record, or that they would receive test results via their PHR rather than from their doctor or medical practice, despite this being a concern that clinicians have [18]. They were supportive of data sharing to help improve service delivery as long as they remained anonymous. However, they were interested in receiving personalised messages and notifications about health risky behaviours or parameters.

Many of our participants were keen to use apps and devices to monitor parameters relevant to their health and wellbeing. Some were already doing so and welcomed a “single place of truth” that they could use to access and share this information. This level of interest in using monitoring for a person-driven preventative approach to health has not previously been described. Developing a PHR that interfaces with apps has several advantages in terms of achieving behavioural change, as it would allow the PHR to specify the information that apps should collect from users. There are several behaviour change techniques [25] that are likely to be particularly appropriate. These include goal setting, developing actions plans, identifying and sourcing social support, and identifying and overcoming barriers.

People receive care and support from different health and social care organisations at different times. Participants found co-ordinating their networks of providers frustrating and saw a role for themselves in bridging communication gaps that can appear at organisational boundaries [26]. Empowering patients to take a more active role in managing this complicated health and care landscape may have a positive impact on health outcomes and wider patient outcomes, such as patient safety. First of all, it may enable people to play a proactive role in optimising their care. Participants in our study saw a role for a PHR in assisting them in routine tasks such as managing their medicines. Other research has identified the strategies that patients use to adhere to their treatment regimens, using strategies such as creating visual cues and developing routines to manage resources [27]. Our findings indicate that a PHR might enhance people’s abilities to better manage health-related tasks, keep accurate records and track progress. Secondly, the increasing prevalence of multi-morbidity means that people may be receiving treatment from different healthcare providers who may not communicate effectively with each other. When care transfers across boundaries of responsibility people are at higher risk of adverse events [28]. A record that is held by the individual would enable them to control information and communicate about the care and treatment they are currently receiving thereby actively involving patients in their own safety [29].

Ensuring that people feel supported to achieve what matters to them is a key measure of success in delivering the NHS vision for personalised, co-ordinated and empowering care and support [30]. Importantly, participants in our study discussed how a PHR would enable them to integrate their self-collected data into the record which could support healthier choices and behaviour. It would also allow people to integrate their own goals and achievements into their record. There is a current drive to make care more personalised and geared to supporting people to recognise their own strengths [31, 32]. Our participants also believed that the PHR may act to balance the dynamic between themselves and their care providers, making the relationships more person-centred and less paternalistic. Our data show that people believe having control of their health records can support their empowerment and create more equal relationships with the professionals they interact with.

The strength of our research is in the wide range of participants we included: they were not limited to a particular, or any, health condition. They included patients and advocates, teens, and people who already log health and wellbeing data. All groups of participants identified advantages of using PHR and recognised how the way in which they use it would change over their lifespan, and accordingly, it would bring different benefits, from prevention, to faster diagnosis and more appropriate treatment. While we know that younger healthy adults are less likely to find a PHR useful [2] our research has shown that teens would use their record in a different way by tracking things that are important to them, such as their weight, physical activity, diet and mood. While previous research has highlighted PHRs can fail because people do not see them as personally relevant [7] our research has highlighted the importance of PHRs being able to interface with apps that individuals *do* find personally relevant. A disadvantage of this research is that participants discussed the PHR without seeing or interacting with one, and while this meant they were more creative about how they could use a PHR, it meant that they did not discuss practical difficulties in using it. We know that usability is a key feature that affects the extent to which people engage with a PHR and so a future PHR would need to be user tested. With good interactivity and user experience a PHR will have a changing function throughout an individual’s lifespan and has the potential to bring major benefits to public health.

## Conclusions

This research has shown that a wide range of potential users would be willing to engage with a PHR if it can work dynamically, with interactive functions and be well-integrated with lifestyle and health apps. To have confidence in a PHR, potential users want control over their personal information, including what is shared, with who and when and they want assurances that that their information is secure and acted upon by professionals who they interact with through the record. A PHR developed with this functionality has the potential to facilitate a more person-driven health and social care system, giving professionals more holistic and detailed insight into an individual’s health behaviours and health risks. It has the potential to reduce patient safety incidents as care transfers across boundaries of responsibility by supporting patients’ active role in their own safety and health outcomes. A PHR could also deliver wide-ranging public health benefits and preventative health interventions through prompting and encouraging users to increase their health-protective behaviours, reduce risks to their health and offer support to self-manage treatment regimens. A PHR could specify the evidence-based behaviour change techniques that existing behaviour change apps should deliver to increase their effectiveness.

## Abbreviations

EHR: Electronic health record
NHS: National Health Service
PHR: Personal health record
